# Supplementary material for: OpenMM 7: Rapid development of high performance algorithms for molecular dynamics
Source: PLoS Comput Biol. 2017 Jul 26;13(7):e1005659. doi: 10.1371/journal.pcbi.1005659 (PMC5549999; doi:10.1371/journal.pcbi.1005659)
Supplement: S1 Source Code — (ZIP) [file pcbi.1005659.s002.zip › OpenMM-7.0.1-Source/docs/api-c++/generated/OpenMM.XmlSerializer.html]

XmlSerializer — OpenMM @OPENMM\_MAJOR\_VERSION@.@OPENMM\_MINOR\_VERSION@ documentation


# XmlSerializer¶

*class* `OpenMM::``XmlSerializer`¶
:   `XmlSerializer` is used for serializing objects as XML, and for reconstructing them again.

    `template <class T>`

    void `serialize`(*const* T \**object*, *const* std::string &*rootName*, std::ostream &*stream*)¶
    :   Serialize an object as XML.

        |  |  |
        | --- | --- |
        | Parameters: |  |

        - **object** – the object to serialize
        - **rootName** – the name to use for the root node of the XML document
        - **stream** – an output stream to write the XML to

    `template <class T>`

    T \*`deserialize`(std::istream &*stream*)¶
    :   Reconstruct an object that has been serialized as XML.

        |  |  |
        | --- | --- |
        | Parameters: |  |

        - **stream** – an input stream to read the XML from

        |  |  |
        | --- | --- |
        | Returns: | a pointer to the newly created object. The caller assumes ownership of the object. |

    `template <class T>`

    T \*`clone`(*const* T &*object*)¶
    :   Clone an object by first serializing it, then deserializing it again. This method constructs the new object directly from the SerializationNodes without first converting them to XML. This means it is faster and uses less memory than making separate calls to `serialize()` and `deserialize()`.

High performance molecular simulation on GPUs


### Navigation

- Core Objects
- Forces
- Integrators
- Extras

©2015, Stanford University and the Authors.
|
Powered by Sphinx 1.3.1
& Alabaster 0.7.7
|
Page source
